# A framework of biomarkers for skeletal muscle aging: a consensus statement by the Aging Biomarker Consortium

**DOI:** 10.1093/lifemedi/lnaf001

**Published:** 2025-01-26

**Authors:** Aging Biomarker Consortium, Ning Huang, Meiling Ge, Xiaolei Liu, Xu Tian, Pengbin Yin, Zhijun Bao, Feng Cao, Ng Shyh-Chang, Biao Dong, Lunzhi Dai, Zhenji Gan, Ping Hu, Jing Qu, Si Wang, Huating Wang, Qian Xiao, Rui Yue, Jirong Yue, Licheng Zhang, Yong Zhang, Hongbo Zhang, Weiqi Zhang, Guang-Hui Liu, Gang Pei, Yong Liu, Dahai Zhu, Birong Dong

**Affiliations:** The Center of Gerontology and Geriatrics and National Clinical Research Center for Geriatrics, West China Hospital, Sichuan University, Chengdu 610041, China; Department of Geriatrics, West China Hospital, Sichuan University, Chengdu 610041, China; The Center of Gerontology and Geriatrics and National Clinical Research Center for Geriatrics, West China Hospital, Sichuan University, Chengdu 610041, China; Department of Geriatrics, West China Hospital, Sichuan University, Chengdu 610041, China; The Center of Gerontology and Geriatrics and National Clinical Research Center for Geriatrics, West China Hospital, Sichuan University, Chengdu 610041, China; Department of Geriatrics, West China Hospital, Sichuan University, Chengdu 610041, China; Bioland Laboratory (Guangzhou Regenerative Medicine and Health Guangdong Laboratory), Guangzhou 510005, China; Department of Orthopedics, Chinese PLA General Hospital, Beijing 100853, China; National Clinical Research Center for Orthopedics, Sports Medicine & Rehabilitation, Beijing 100853, China; Department of Geriatrics, Huadong Hospital, Shanghai Medical College, Fudan University, Shanghai 200040, China; Department of Cardiology, The Second Medical Centre, Chinese PLA General Hospital, National Clinical Research Center for Geriatric Diseases, Beijing 100853, China; Key Laboratory of Organ Regeneration and Reconstruction, State Key Laboratory of Stem Cell and Reproductive Biology, Institute of Zoology, Chinese Academy of Sciences, Beijing 100101, China; Institute for Stem Cell and Regeneration, Chinese Academy of Sciences, Beijing 100101, China; University of Chinese Academy of Sciences, Beijing 100101, China; Beijing Institute for Stem Cell and Regenerative Medicine, Beijing 100101, China; National Clinical Research Center for Geriatrics and State Key Laboratory of Biotherapy, West China Hospital, Sichuan University, Chengdu 610041, China; Sichuan Real and Best Biotech Co., Ltd., Chengdu 610041, China; National Clinical Research Center for Geriatrics and State Key Laboratory of Biotherapy, West China Hospital, Sichuan University, Chengdu 610041, China; State Key Laboratory of Pharmaceutical Biotechnology and MOE Key Laboratory of Model Animal for Disease Study, Model Animal Research Center, Department of Spine Surgery, Nanjing Drum Tower Hospital, The Affiliated Hospital of Nanjing University Medical School, Nanjing University Medical School, Nanjing University, Nanjing 210061, China; Spine Center, Xinhua Hospital Affiliated to Shanghai Jiao Tong University School of Medicine, Shanghai 200072, China; Guangzhou Laboratory, Guangzhou 510005, China; Key Laboratory of Biological Targeting Diagnosis, Therapy and Rehabilitation of Guangdong Higher Education Institutes, the Fifth Affiliated Hospital of Guangzhou Medical University, Guangzhou 510005, China; The Tenth People’s Hospital Affiliated to Tongji University, Shanghai 200072, China; State Key Laboratory of Stem Cell and Reproductive Biology, Institute of Zoology, Institute for Stem Cell and Regeneration, Institute for Stem Cell and Regenerative Medicine, University of Chinese Academy of Sciences, Beijing 100101, China; Beijing Municipal Geriatric Medical Research Center, Xuanwu Hospital, Capital Medical University, Beijing 100053, China; Aging Translational Medicine Center, International Center for Aging and Cancer, Xuanwu Hospital, Capital Medical University, Beijing 100053, China; Advanced Innovation Center for Human Brain Protection, and National Clinical Research Center for Geriatric Disorders, Xuanwu Hospital Capital Medical University, Beijing 100053, China; Li Ka Shing Institute of Health Sciences, The Chinese University of Hong Kong, Hong Kong 999077, China; Department of Orthopedics and Traumatology, Prince of Wales Hospital, Li Ka Shing Institute of Health Sciences, The Chinese University of Hong Kong, Hong Kong 999077, China; Department of Geriatrics, The First Affiliated Hospital of Chongqing Medical University, Chongqing 400016, China; Shanghai Key Laboratory of Signaling and Disease Research, Frontier Science Center for Stem Cell Research, School of Life Sciences and Technology, Institute for Regenerative Medicine, Shanghai East Hospital, Tongji University, Shanghai 200092, China; The Center of Gerontology and Geriatrics and National Clinical Research Center for Geriatrics, West China Hospital, Sichuan University, Chengdu 610041, China; Department of Geriatrics, West China Hospital, Sichuan University, Chengdu 610041, China; Department of Orthopaedic Trauma, the Fourth Medical Center, National Clinical Research Center for Orthopaedics & Sports Rehabilitation in China, Chinese PLA General Hospital, Beijing 100853, China; Bioland Laboratory (Guangzhou Regenerative Medicine and Health Guangdong Laboratory), Guangzhou 510005, China; The State Key Laboratory of Medical Molecular Biology, Institute of Basic Medical Sciences, Chinese Academy of Medical Sciences and School of Basic Medicine, Peking Union Medical College, Beijing 100005, China; Center for Stem Cell Biology and Tissue Engineering, Key Laboratory for Stem Cells and Tissue Engineering, Ministry of Education, Sun Yat-sen University, Guangzhou 510080, China; The SYSU-YSG Joint Laboratory for Skin Health Research, Sun Yat-sen University, Guangzhou 510080, China; Advanced Medical Technology Center, The First Afiliated Hospital, Zhongshan School of Medicine, Sun Yat-sen University, Guangzhou 510080, China; CAS Key Laboratory of Genomic and Precision Medicine, Beijing Institute of Genomics, Chinese Academy of Sciences and China National Center for Bioinformation, Beijing 100101, China; State Key Laboratory of Membrane Biology, Institute of Zoology, Institute for Stem Cell and Regeneration, University of Chinese Academy of Sciences, Chinese Academy of Sciences, Beijing 100101, China; The Collaborative Innovation Center for Brain Science, School of Life Sciences and Technology, Tongji University, Shanghai 200070, China; Hubei Key Laboratory of Cell Homeostasis, College of Life Sciences; TaiKang Center for Life and Medical Sciences; the Institute for Advanced Studies; Frontier Science Center for Immunology and Metabolism, Wuhan University, Wuhan 430072, China; Bioland Laboratory (Guangzhou Regenerative Medicine and Health Guangdong Laboratory), Guangzhou 510005, China; The State Key Laboratory of Medical Molecular Biology, Institute of Basic Medical Sciences, Chinese Academy of Medical Sciences and School of Basic Medicine, Peking Union Medical College, Beijing 100005, China; The Center of Gerontology and Geriatrics and National Clinical Research Center for Geriatrics, West China Hospital, Sichuan University, Chengdu 610041, China; Department of Geriatrics, West China Hospital, Sichuan University, Chengdu 610041, China

## Abstract

The skeletal muscle is an important organ for movement and metabolism in human body, and its physiological aging underlies the occurrence of muscle atrophy and sarcopenia. China has the largest aging population in the world and is facing a grand challenge with how to prevent and treat skeletal muscle aging-related diseases. To address this difficult problem, the Aging Biomarker Consortium (ABC) of China has reached an expert consensus on biomarkers of skeletal muscle aging by synthesizing literatures and insights from scientists and clinicians. This consensus attempts to provide a comprehensive assessment of biomarkers associated with skeletal muscle aging, and proposes a systematic framework to classify them into three dimensions: functional, structural, and humoral. Within each dimension, the experts recommend clinically relevant biomarkers for skeletal muscle aging. This consensus aims to lay the foundation for future research on skeletal muscle aging, facilitating precise prediction, diagnosis, and treatment of skeletal muscle aging and sarcopenia. It is anticipated to make significant contributions to healthy aging of skeletal muscle in the elderly population in China and around the world as well.

## Introduction

With advancing age, skeletal muscle undergoes progressive changes in terms of structure and function, leading to sarcopenia, muscle atrophy, and an increased risk of falls and mortality [1–3]. Skeletal muscle aging severely affects the quality of life and can even lead to systemic functional decline in multiple organ systems. According to the National Bureau of Population Statistics, the elderly people over 60 years in China have reached 297 million [4]. Moreover, research show that a prevalence of sarcopenia at over 10%, which has become a significant health and public health issue, posing a serious threat to the life and health of the elderly [3]. Therefore, early detection, early warning, early prevention, and early treatment of skeletal muscle aging are of great importance for addressing the aging population in China.

Skeletal muscle aging refers to the structural changes and functional decline that occur in skeletal muscle with aging, including biological aging and pathological aging [1]. Pathological manifestations include the reduction of skeletal muscle mass, the decrease of myofiber cross-sectional area, the abnormalities of myofiber microscopic structure, the fat deposition in skeletal muscle, the fibrosis and hyperplasia of skeletal muscle, and the changes of myofiber types [5, 6]. Functional changes include the impairment of muscle contraction and regeneration function, the reduction of skeletal muscle secretion function, and dysregulation of skeletal muscle metabolism [7–9]. Therefore, identifying biomarkers that accurately reflect the degree of skeletal muscle aging will help to precisely assess the functional state of skeletal muscle, predict the risk of skeletal muscle aging, and take timely individualized intervention approaches to prevent and treat skeletal muscle aging.

On 6 July 2024, the Chinese Aging Biomarker Consortium (ABC) [10–12] convened a workshop at the National Clinical Research Center of Geriatric Diseases, West China Hospital, Sichuan University to identify the relevant biomarkers for skeletal muscle aging based on the literature and evidence-based research. The expert consensus, based on peer-reviewed studies and evidence-based medicine [13–17], recommended biomarkers that can indicate the degree and rate of skeletal muscle aging, predict the risk of skeletal muscle aging, and provide a scientific evaluation system for slowing down and intervening in aging and its related diseases.

## Recommended methodology for biomarkers for skeletal muscle aging

We searched for skeletal muscle aging relevant research articles published before May 2024 in databases, such as MEDLINE, PubMed, and the Cochrane Library. The members of ABC collaborated to summarize a list of current biomarkers associated with skeletal muscle aging based on the existing research evidence. The list of search keywords in this consensus is provided in the Supplementary Materials (Search keywords sheet).

The presentation of the level of recommendation and level of evidence in this consensus follows the internationally accepted conventions. The level of evidence and strength of recommendations, which are detailed in Table 1.

**Table 1.** Class of recommendations and level of evidence

Class (strength) of recommendation	Level (quality) of evidence
**CLASS I (strong) benefit >>> risk**	**Level A**
**Suggested phrases for writing recommendation** Recommended/Indicated Evidence and/or general agreement that a given treatment or procedure is beneficial, useful and effective	• Data derived from multiple randomized clinical trials or meta-analyses
**CLASS IIa (moderate) benefit >> risk**	**Level B**
**Suggested phrases for writing recommendation** Should be considered Weight of evidence/opinion is in favour of usefulness/efficacy	Data derived from a single randomized clinical trial or large non-randomized studies
**CLASS IIb (weak) benefit ≥ risk**	**Level C**
**Suggested phrases for writing recommendation** May be considered Usefulness/efficacy is less well established by evidence/opinion	Consensus of expert opinion, and/or small studies, retrospective studies, registries
**CLASS III (strong) risk > benefit** **Suggested phrases for writing recommendation** Not recommended Evidence/general agreement that the given treatment/procedure is not useful/effective and sometimes maybe harmful	**Note:** COR and LOE are determined independently (any COR may be paired with any LOE). COR, Class of Recommendation; LOE, Level of Evidence.

## Classification and clinical application of skeletal muscle aging biomarkers

**Figure 1.**
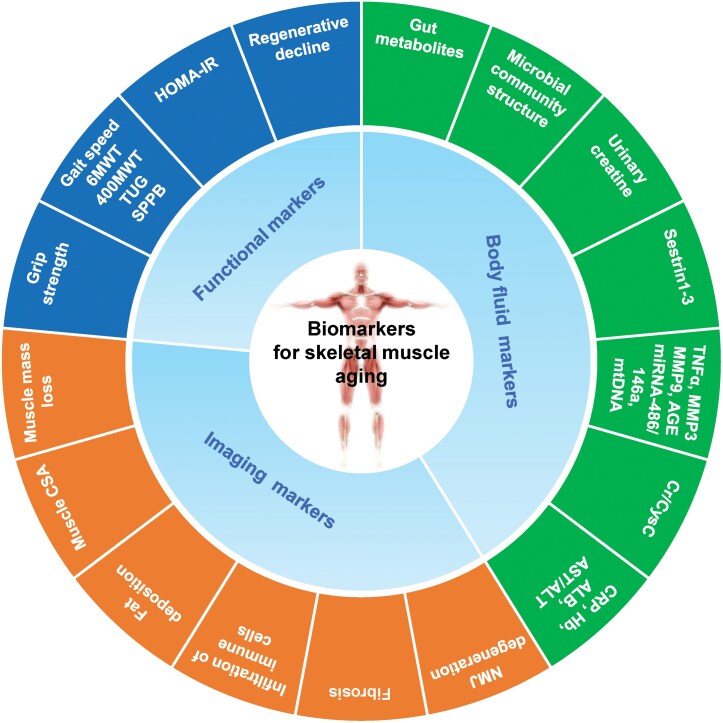
**Biomarker framework for skeletal muscle aging.** The proposed framework for the assessment of skeletal muscle aging consists of three dimensions biomarkers: functional, imaging, and body fluid. These biomarkers are expected to be widely used in routine clinical practice. Specially, it is important to emphasize that further validation is needed to assess the validity of these biomarkers in the assessment of skeletal biological aging. Abbreviations involved in the Fig. 1 refer to Table 2.

Skeletal muscle aging involves multi-dimensional and multi-level changes in molecules, cells, organs, and population [10, 11]. Skeletal muscle aging biomarkers refer to those that can accurately predict the “actual age” of skeletal muscle, structure, and function, which can be used to assess the degree of skeletal muscle aging and evaluate the effectiveness of interventions. Considering the accessibility and convenience of clinical operations, this consensus delineates the screening of skeletal muscle aging biomarkers from the dimensions of function, structure, and body fluids (Fig. 1).

**Table 2.** Abbreviations in this consensus

Abbreviations	Full name of abbreviation
25(OH)D	25-hydroxyvitamin D
5-HT	5-hydroxytryptamine
6MWD	Distance covered in 6 minutes of walking
6MWT	6-minute walk test
ABC	Aging Biomarker Consortium
AGEs	Advanced glycation end products
AI	Artificial intelligence
AKT	Protein kinase B
ALB	Albumin
ALT	Alanine transaminase
AST	Aspartate transaminase
AUC	Area under the curve
AWGS	Asian Working Group for Sarcopenia
BA	Bile acids
BCAAs	Branched-chain amino acids
BDNF	Brain-derived neurotrophic factor
BIA	Bioelectrical impedance analysis
BL986	*Bifidobacterium longum* BL986
BMI	Body Mass Index
cAMP	Cyclic adenosine monophosphate
ccf-mtDNA	Circulating cell-free mitochondrial DNA
CD47	Cluster of differentiation 47
CKD	Chronic kidney disease
Cr	Creatinine
Cr/CysC	Serum creatinine/Cystatin C
CRP	C-reactive protein
CSA CT	Cross-sectional area Computed tomography
CXCL8	CXC chemokine ligand 8
CysC	Cystatin C
D2	Type 2 iodothyronine deiodinase
dEMG	Dynamic electromyography
DHEA	Dehydroepiandrosterone
DMD	Duchenne muscular dystrophy
dSTORM	Direct stochastic optical reconstruction microscopy
DTI	Diffusion tensor imaging
DXA	Dual energy X-ray absorption
ECM	Extracellular matrix
EMG	Electromyography
EWGSOP2	European Working Group on Sarcopenia in Older People 2
FAPs	Fibroblast/adipogenic progenitors
GLUT4	Glucose transporter 4
GSH	Glutathione
Hb	Hemoglobin
HOMA-IR	Homeostasis model assessment of insulin resistance
IAA	Indole-3-acetic acid
IAAld	Indole-3-acetaldehyde
IAld	Indole-3-aldehyde
IGF-1	Insulin-like growth factor 1
IL-1	Interleukin-1
IL-18	Interleukin-18
IL-8	Interleukin-8
IPA	Indole-3-propionic acid
IR	Insulin resistance
IRAK-1	IL-1R-associated kinase
Kyn	Kynurenine
LC122	*Lactobacillus bulgaricus* LC122
LPPS23	*Lactobacillus paracasei* PS23
LPS	Lipopolysaccharide
MHD	Maintenance hemodialysis
MMP-3	Matrix metalloproteinase-3
MMP-9	Matrix metalloproteinase-9
MPS	Muscle protein synthesis
MRI	Magnetic resonance imaging
mtDNA	Mitochondrial DNA
mTOR	Mammalian target of rapamycin
MuSCs	Muscle stem cells
NMJ	Neuromuscular junction
OLP-01	*Bifidobacterium longum* OLP-01
PAI-1	Plasminogen activator inhibitor-1
Pax7	Paired box 7
PI3K	Phosphatidylinositol3 kinase
ROS	Reactive oxygen species
S6K1	Ribosomal protein S6 kinase
SB	*Saccharomyces boulardii*
SCFAs	Short-chain fatty acids
sEMG	Surface electromyography
SESNs	Sestrins
SFEMG	Single fiber electromyography
SPPB	Short Physical Performance Battery
STED	Stimulated emission depletion
TGF-β	Transforming growth factor-β
TIMP1	Tissue inhibitor of metalloproteinase 1
TNF-α	Tumor necrosis factor-α
TRAF6	TNF receptor-associated factor 6
Trp	Tryptophan
TUG	Timed up and go
US	Ultrasound
VDR	Vitamin D receptor
WCHAT	Western China Health and Aging Trend

### Functional markers

#### Mobility function decline

The human body contains more than 600 muscles, accounting for about 40% of its total weight, including skeletal muscle, myocardium, and smooth muscle. Among them, skeletal muscle controlled by human consciousness and is also known as “voluntary muscles.” Skeletal muscles play an important role in maintaining normal bodily functions. From a mechanical perspective, the main function of skeletal muscles is to convert chemical energy into mechanical energy to generate strength and power, thereby maintaining body posture, normal bodily movements, and daily life functions [18].

Skeletal muscles generally span one or more joints and can be attached to one or more bones at each end, as well as ligaments, fascia, and skin. Muscle contraction pulls the attached bones, producing motion with the joints as the fulcrum, or directly pulling the skin to produce motion.

Skeletal muscles usually attach to bones and have two attachment sites. One attachment site is called the starting point of skeletal muscle. This starting point can be a small but visible point on the bone, or a large area covering a part of the bone. The starting point is usually on the bone closest to the core of the body. The other site is called the endpoint. The endpoint of the muscle usually connected to the tendon, which runs through the joint to make the muscle function. Skeletal muscle may have more than one starting point or endpoint, and in this case, skeletal muscle is divided into segments called the head [19, 20].

During the aging process, the physical function of the older adults always is impaired or reduced, such as grip strength decline, slower walking speed, or impaired balance ability, which is correlated with the quality and strength of the skeletal muscles they control. For example, (i) the quadriceps femoris, consisting of the rectus femoris, lateral femoris, medial femoris, and intermediate femoris muscles, is innervated by the femoral nerve and the main muscle maintains the upright, walking, running, and jumping of the human body. Its function is to extend the knee and bend the hip. When the quadriceps femoris contracts, it pulls the patella and patellar ligament to complete the knee extension movement; (ii) the biceps, brachialis, and brachioradialis muscles, which are closely related to grip strength, play a key role in elbow flexion, forearm extension and movement, flexion, and rotation of the elbow joint; (iii) the muscle groups closely related to the balance include the iliopsoas muscle, as well as the core muscle groups rectus abdominis and latissimus dorsi, which control hip joint backward movement, trunk forward flexion, shoulder joint and arm backward movement, respectively and are crucial for maintaining balance ability.

#### Decreased muscle contraction function

The types of muscle contraction can be roughly divided into isometric contraction, isotonic contraction, and isokinetic contraction. Isometric contraction, also known as stationary contraction, refers to the significant increase in muscle strength during muscle contraction, but the muscle length remains basically unchanged and does not produce joint movement contraction. It is commonly used in daily life and work to maintain specific positions and postures. Isotonic contraction refers to the contraction of muscles during muscle contraction, where the muscle strength remains basically unchanged but the muscle length changes, causing joint movement contraction, which can be divided into centripetal contraction and centrifugal contraction. Isokinetic contraction refers to the form of muscle contraction in which the speed (angular velocity) remains unchanged during muscle contraction and is completed with the help of an isokinetic device. It is not a natural contraction form of muscles but a method of muscle strength evaluation and training.

##### Isokinetic muscle strength testing

As a new muscle function testing and evaluation method, isokinetic muscle strength testing has been widely used in clinical and basic research of rehabilitation medicine and sports medicine. The objectivity and reliability of isokinetic muscle strength testing technology have been studied by many domestic and foreign scholars [21]. Isokinetic muscle strength testing conforms to the changes in the cross-sectional area of exercise muscles, which can not only effectively evaluate muscle strength, but also reflect muscle strength changes that cannot be observed by manual muscle strength testing.

##### Grip strength

Grip strength is commonly used to quantify the muscle strength. Handheld hydraulic grip strength meter—JAMAR—is the gold standard for grip strength measurement [22]. When measuring the grip strength, the subjects should sit, bend the elbow, and place the forearm on the chair arm, and both hands for three times were measured, with a 10-second interval and then record the maximum value and dominant hand. If using a spring grip dynamometer, it is necessary to take a stand position and extend the elbow. If older adults cannot stand, they can also be measured while sitting [23, 24]. According to the Asian Working Group for Sarcopenia (AWGS) consensus standard in 2019, grip strength < 28 kg for male and grip strength < 18 kg for female is defined as muscle strength decline. The European Working Group on Sarcopenia in Older People 2 (EWGSOP2) recommends the grip strength < 27 kg for male and grip strength < 16 kg for female as the criteria for muscle strength decline [25]. Therefore, grip strength has become an important indicator of skeletal muscle contraction function.

#### Physical activity decline

##### Gait speed test

The subject completes a 4-m flat walk at normal speed and records the time it takes to cross the starting and ending lines. Repeat the measurement once and take the shortest time as the test result. The completion time is divided into 4.82 s, 6.20 s, and 8.71 s and is counted as 4, 3, 2, and 1 point, respectively [26, 27]. If it cannot be completed, it is counted as 0 points. EWGSOP2 recommends a 4-m gait speed test, and speed ≤ 0.8 m/s indicates physical function decreased. However, AWGS 2019 recommends the 6-m gait speed test, using the same measurement method as a 4-m. If a 6-m gait speed < 1.0 m/s, it is diagnosed as slowed gait speed [23].

The 6-minute walk test (6MWT) is a simple endurance test that evaluates activity ability and functional reserve (Table S1). Participants walk back and forth along a prescribed 30-m walk route, record the distance covered in 6 minutes of walking (6MWD), as well as blood pressure, heart rate, blood oxygen saturation, and Borg index scores. Expert consensus recommends a predictive formula suitable for the elderly population in China, which is: male, 6MWD (m) = 233.205 + [4.31 × height (cm)] − (4.01 × age + 6.14 × BMI); female, 6MWD (m) = 285.011 + [1.041 × height (cm)] − (0.678 × age + 2.189 × BMI). Participants with 6MWD > 450 m are normal, 301–450 m are mild abnormal, 151–300 m are moderate abnormal, and < 150 m are severe abnormal. The diagnostic criteria for AWGS sarcopenia recommend a gait speed test of 6MWT, with gait speed of ≤ 1.0 m/s indicating physical function decreased [23].

##### Four hundred-meter walking test (400MWT)

The subject turns back 10 times at a normal speed on a 20-m long flat. The subject’s blood pressure and 30-second pulse are measured before the test. After the fourth turn back, the subject is asked to report their fatigue level and record the Borg index score. During the test, the subject is allowed to rest for 30 seconds while standing, with no limit on the number of times. If the subject cannot continue walking or needs to sit down to rest after resting for 60 seconds, the test must be stopped. The time for walking 400 m is recorded, or the walking distance of the subject who cannot complete it within 15 minutes is recorded. At the end of the test, the subject’s sitting blood pressure and 30-second pulse and Borg index score are measured, and the number, time, and reason for rest are recorded [26]. The diagnosis criteria sarcopenia in EWGSOP2, the inability to complete the 400-m walking test or completion time ≥ 6 minutes is defined as decreased physical function.

##### Timed up and go (TUG) test

TUG is mainly used to evaluate the activity and balance abilities of elderly people (Table S2). During the test, participants are asked to stand up from their seats, walk straight forward for 3 m, and then turn back to their seats to record the completion time. < 10 s is normal, 10–19 s are mild abnormalities, 20–29 s are moderate abnormalities, and ≥ 30 s are severe abnormalities [28]. EWGSOP 2 defines sarcopenia using TUG ≥ 20 s as decreased physical function [25]. AWGS 2019 not recommended TUG test since it may be influenced by multiple factors.

The short physical performance battery (SPPB) includes (i) Gait speed test. (ii) Balance test: (a) side-by-side stand: stand with both feet together, side-by-side for about 10 seconds; (b) semi-tandem stand: stand with the side of the heel of one foot touching the big toe of the other foot for about 10 seconds; (c) tandem stand: stand with the heel of one foot in front and touching the toes of the other foot for about 10 seconds. The first two positions held for ≥ 10 s for 1 point, < 10 s or not attempted for 0 point; the third position held for ≥ 10 s for 2 points, 3–9.99 s for 1 point, and < 3 s or not attempted for 0 point. (iii) Repeated chair stand test: Stand up from a chair five times without using the arms, completes 5 sit up tests at the fastest speed their body can tolerate, and records the completion time. If ≤ 11.19 s for 4 points; 11.20–13.69 s for 3 points; 13.70–16.69 s for 2 points; > 16.7 s for 1 point; if > 60 s or cannot completed for 0 points. The total score of SPPB ranges from 0 to 12 points, with higher scores indicating poorer physical function. AWGS 2019 defines physical function impaired as SPPB ≤ 9 points or repeated chair test ≥ 12 s, while EWGSOP2 defines physical dysfunction as SPPB ≤ 8 points. The detailed scoring criteria for SPPB are shown in the following figure (Table S3).

##### Balance assessment

Single leg stand time test: It is a simple method of measuring balance ability. Before the test, the subject selects one leg as the supporting leg, and then lifts the other leg from a flat floor with their eyes open. During the test, the lifted leg cannot rest on the supporting leg. When the lifted leg touches the supporting leg, touches the floor, or successfully balances for 60 seconds, the test ends. Record the time the subject stays standing on one leg with their eyes open. The shorter maintenance time means the worse balance function. Some studies have also used single leg stand time to evaluate balance function and developed scoring standards suitable for the older adults in China. It was found that the balance of the human body reaches its peak around the age of 20, and thereafter it shows a gradually decreasing trend. However, the single leg stand test is more effective than SPPB. The balance test in TUG is relatively difficult, and the test process should be based on the subjects’ tolerance to ensure the safety during the process.

Other methods also could be used for physical function assessment, such as Berg balance scale, Tinetti gait and balance test scale, and so on. To reduce the influence of subjective factors, the digital senile physical function assessment system has been used to quantify the physical function. With the progress of artificial intelligence (AI), it is expected to be further used in clinical practice. Therefore, gait speed test, 6MWT, 400MWT, repeated chair stands test, SPPB, and balance test all could be used to evaluate the skeletal muscle function status.

#### Dysfunction of glucose metabolism

Skeletal muscle is an essential organ in glucose metabolism, and the decline in glucose metabolic function is an important characteristic of skeletal muscle aging. With advancing aging, a variety of factors contribute to the reduction of glucose metabolism, such as impaired insulin signaling, alterations in muscle fiber types, mitochondrial dysfunction, reduced muscle mass and function, increased inflammation and oxidative stress, endocrine imbalances, and lifestyle factors [29]. Moreover, there are changes in the expression and activity of enzymes related to glucose metabolic pathways in skeletal muscle. For instance, it has been reported that the glucose uptake capacity is reduced in older men (65–70 years old) compared to younger men (30 years old), which is associated with decreased activity of key enzymes in the glucose metabolic pathway, such as glucose transporter 4 (GLUT4) [30, 31]. Concurrently, the activity of the insulin pathway is reduced in the skeletal muscle of the elderly, such as the decreased activity of the PI3K/AKT pathway in the vastus lateralis muscle of older individuals [32, 33]. Additionally, several studies have reported a positive correlation between the insulin resistance index (HOMA-IR) and sarcopenia [34–37]. Therefore, monitoring the (HOMA-IR) can serve as an indicator to evaluate the status of skeletal muscle aging.

#### Decreased regenerative function

The regenerative capacity of skeletal muscle gradually decreases, which is associated with a reduction in the number and function of skeletal muscle stem cells (MuSCs) [38]. Skeletal muscle is derived from the proliferation and differentiation of muscle stem cells located on the basal lamina of muscle fibers. MuSCs play a central role in the maintenance, growth, repair, and regeneration of skeletal muscle. With aging, MuSCs occurs changes in both number decline and cellular subset ratio variation [8, 39]. Several research groups have identified functionally distinct MuSC subtypes based on the expression levels of Pax7, CD47, GSH, and other markers in MuSCs, and the proportions of these characteristic cell subsets change during the aging process. For example, it has been reported that the Pax7hi MuSCs, which have higher stemness characteristic, significantly decrease in old mice, while the proportion of functionally impaired CD47hi MuSCs and GSHlow MuSCs, which have reduced proliferative capacity, are more prone to apoptosis [40–42]. These studies indicate that the change of MuSC cell subsets are important characteristics of the decline in skeletal muscle regenerative capacity. Based on these findings, the regenerative capacity of skeletal muscle can be assessed by analyzing the proportions of MuSC cell subsets (Pax7hi, CD47hi, GSHlow) using muscle biopsies, combined with flow cytometry sorting and single-cell sequencing in the future.

#### Recommendations

(1) Grip strength decline with aging and can be used as a marker for evaluating the contractile capacity of skeletal muscles (Level A evidence, Class I recommendation).
(2) Gait speed test, the 6MWT, the 400MWT, TUG, and the SPPB can be used as functional markers for assessing age-related sarcopenia. (Level A evidence, Class I recommendation).
(3) Insulin sensitivity decreases with age and can be used as a functional marker for predicting skeletal muscle aging by measured HOMA-IR (Level A evidence, Class IIa recommendation).
(4) The reduction of satellite cell numbers and the alteration of satellite cell subsets with aging can indicate the decline of skeletal muscle regenerative capacity (Level B evidence, Class III recommendation).

### Imaging markers

Owing to its intuitive, quantitative and easy-to-operate characteristics, imaging has been widely used in the diagnosis, efficacy evaluation, and prognostic assessment of skeletal muscle degenerative diseases and age-related disorders. Imaging examination can dynamically monitor the changes of skeletal muscle quality or composition, and provide important clues for the early warning and diagnosis of skeletal muscle aging and related diseases.

#### Decreased skeletal muscle mass

Human aging is accompanied by progressive decline of skeletal muscle mass. Generally, skeletal muscle mass is peaked in young adults (about 30 years old) and gradually decreased with age. The decline in total skeletal muscle mass begins at the age of 30–40 years, with lower extremity muscles decreasing at a rate of about 0.7%–0.8% per year [3, 43, 44]. Therefore, skeletal muscle mass decline has been proposed as a biomarker of skeletal muscle aging. It can be used as an indicator for the early warning and diagnosis of skeletal muscle aging, which is helpful for early prevention of aged-related degenerative skeletal muscle disease. Clinically, the decrease of skeletal muscle mass has been used to predict the risk of sarcopenia.

Skeletal muscle mass could be measured by dual energy X-ray absorption (DXA), magnetic resonance imaging (MRI), computed tomography (CT), bioelectrical impedance analysis (BIA), ultrasound (US), photoacoustic imaging (PAI), and other imaging methods [3, 45, 46]. The most effective procedure to date is the use of DXA, which estimates lean mass. BIA, CT, and MRI can also be applied according to the actual situation. In Asian population, DXA detection of skeletal muscle mass is less than 7.0 kg/m^2^ (male), 5.4 kg/m^2^ (female) or BIA detection of skeletal muscle mass is less than 7.0 kg/m^2^ (male), 5.7 kg/m^2^ (female), which have been proposed as an important reference for the diagnosis of sarcopenia [23].

#### Decrease in the number and cross-sectional area of muscle fibers

The decrease of skeletal muscle mass during aging is mainly due to the reduction in the number and area of muscle fibers [47]. A study on the biopsy of the vastus lateralis muscle found that the muscle fiber number at the age of 90 years was only half of that of the young [48]. The apoptosis of muscle fibers in the elderly was significantly higher thanthat in the young, which may be one of the reasons for the decrease in the number of muscle fibers [49]. During aging, total cross-sectional area of skeletal muscle decreased significantly. Ultrasound, CT, DXA, MRI and other imaging methods can measure the cross-sectional area, muscle fiber density, and dynamically monitor the changes in the number and area of muscle fibers during skeletal muscle aging. Ultrasound analysis demonstrated that the cross-section area of quadriceps femoris in the elderly was 25%–35% smaller than that in the young [50]. CT scan also found that the skeletal muscle area of the elderly was 28%–36% less than that of the young [51]. Therefore, the reduction in the number and area of muscle fibers can be used as a marker for evaluating skeletal muscle aging.

#### Fiber type alternation

Skeletal muscle is composed of different types of muscle fibers. According to contraction and metabolic features, they can be characterized into slow-twitch oxidative type (type I), fast-twitch oxidative type (type IIA), and fast-twitch glycolytic type (type IIX). Skeletal muscle fiber composition has plasticity, which is affected by age, innervation, mechanical load, energy supply, and other factors. After 70 years of age, the cross-sectional area of type I muscle fibers decreased by 15%–20%, and that of type II muscle fibers decreased by 40% [52]. During skeletal muscle aging, the number of type II muscle fibers decreased significantly [53, 54]. Type II muscle fibers are prone to undergo apoptosis, which may be one of the reasons for the significant reduction of type II muscle fibers [55]. During aging, motor unit remodeling occurs, fast muscle fibers lose innervation, and nerve axons that dominate slow muscle fibers resume innervation, which might be another reason for a significant reduction of type II muscle fibers during skeletal muscle aging. It is worth to note that the muscle fiber type is actually regulated by multiple factors. For example, muscle injury can lead to muscle fiber type alternation, which is similar to the change of muscle fiber types caused by aging. Therefore, muscle fiber type alternation is not recommended as a marker for predicting skeletal muscle aging.

#### Alternations in skeletal muscle composition

##### Increased fat deposition

The deposition of intramuscular fat in skeletal muscle increases with age [54, 56], which is tightly correlated with the risk of skeletal muscle mass loss, skeletal muscle strength decline, and insulin resistance. Dysregulation of fatty acids metabolism is attributed to accumulation of lipid droplets in skeletal muscle [57]. Obesity and inflammation significantly affect fat deposition in skeletal muscle [58]. In addition, fat deposition is also associated with the increased differentiation potential of the adipocyte precursor cells in skeletal muscle during aging. MRI and CT can quantitatively analyze the composition of skeletal muscle and evaluate the degree of fat deposition during skeletal muscle aging [59].

##### Abnormal infiltration of immune cells

Resident immune cells play an important role in regulating inflammatory response and skeletal muscle regeneration. During aging, the reduced recruitment of Treg cells to injured muscle might be attributed to ineffectual repair in aged skeletal muscle [60]. The polarization of macrophages, from pro-inflammatory M1 type to anti-inflammatory M2 type, is crucial for the repair of skeletal muscle injury [61]. MRI is highly sensitive in detecting abnormal signal intensity in skeletal muscle and can recognize immune cell infiltration [62]. In addition, PET imaging can also be used to detect immune cell infiltration via visualizing cell surface markers [63].

##### Fibrosis

Fibrosis is characterized by the abnormal accumulation of collagen and other extracellular matrix components in skeletal muscle, which leads to alternation of muscle stiffness and physiological function during aging. Fibroblast/adipogenic progenitors (FAPS) play a key role in the repair of acute muscle injury, but in chronic injury or myopathy settings, FAPs promote fibrosis and adipogenesis, and eventually impair structure and function of skeletal muscle [64]. The formation of fibrosis is also related to a variety of factors, including inflammatory response, immune cell activation, and the release of transforming growth factor-β [65, 66]. CT and MRI can quantitatively detect skeletal muscle fibrosis [67] and evaluate the degree of skeletal muscle fibrosis during aging. Diffusion tensor imaging (DTI) technology can distinguish healthy muscle and fibrotic muscle tissue by analyzing the anisotropy of H_2_O molecular diffusion. Therefore, using DTI, more accurate information can be obtained in the detection and evaluation of skeletal muscle fibrosis [68].

#### Alternations in innervation

##### Motor neuron degeneration and motor unit remodeling

During aging, the alternations in innervation of skeletal muscle include motor neuron degeneration, neuromuscular junction changes, and motor unit remodeling [69, 70]. Age-related motor unit remodeling involves the denervation of fast-twitch type II muscle fibers, and the reinnervation of nerve axons innervating slow muscle fibers, which lead to altering the proportion of skeletal muscle fiber types and decline of muscle strength. The cross-sectional area and mass of denervated skeletal muscle decreased significantly [71]. After long-term denervation, the subcellular structure of skeletal muscle fibers altered significantly, including swelling nucleus with prominent nucleolus, reduced number of mitochondria, mitochondrial fragmentation, expansion of sarcoplasmic reticulum, excessive enlargement of the terminal cistern, and increased calcium release. Those changes directly affected the excitation contraction coupling of skeletal muscle and performance of physical activity. In addition, the denervation leads to gradually reduce the number of motor end plates [72].

##### Neuromuscular junction degeneration

The neuromuscular junction (NMJ) is the site where the motor neuron makes contact with the skeletal muscle fiber’s membrane (sarcolemma), also known as neuromuscular synapse, which plays an important role in transmitting neuronal signals to skeletal muscle and causing skeletal muscle contraction. During aging, NMJ degenerates, leading to unstable connections between neurons and skeletal muscle fibers, affecting the efficiency of nerve impulse conduction and muscle contraction [73]. The axon terminals of NMJ presynaptic neurons exhibit abnormal thinning or expansion and reduction in the number of synaptic vesicles during aging, which directly affect the efficiency of signal transmission [74]. Denervation leads to dysregulation of the metabolic activity and the contractile function of skeletal muscle.

Electromyography (EMG), as a non-invasive technique that measures the electrical activity of muscles, have been used to monitor the action potentials generated by motor neurons and evaluate nerve impulse conduction and muscle contraction. Surface electromyography (sEMG), also known as dynamic electromyography (dEMG), records the bioelectrical signals during the activity of the neuromuscular system from the muscle surface, and specifically examines the neuromuscular functional state. As a new high-resolution electrophysiological technology, single fiber electromyography (SFEMG) can more accurately evaluate the stability of neuromuscular transmission and the functional state of NMJ, and provide a more reliable and accurate diagnosis for skeletal muscle diseases [75]. In addition, super-resolution microscopy, such as direct stochastic optical reconstruction microscopy (dSTORM) and stimulated emission depletion (STED), can observe the synaptic protein distribution pattern of NMJ [76, 77]. These microscopic pathological measurements will provide valuable molecular imaging markers for the degeneration of NMJ during skeletal muscle aging.

#### Recommendations

(1) Skeletal muscle mass can be an imaging marker to predict skeletal muscle aging, and its decline suggests that skeletal muscle aging may be possible (Level A evidence, Class I recommendation).
(2) Decrease in the number and cross-sectional area of muscle fibers can be an imaging marker to predict skeletal muscle aging, and its presence suggests possible skeletal muscle aging (Level A evidence, Class I recommendation).
(3) Increased fat deposition can be used as a marker to predict skeletal muscle aging (Level B evidence, Class IIb recommendation).
(4) Fibrosis can be used as an imaging marker to predict skeletal muscle aging, and its presence suggests possible skeletal muscle aging (Level C evidence, Class IIb recommendation).
(5) Neuromuscular junction degeneration can be used as an imaging marker to predict skeletal muscle aging, which suggests that skeletal muscle aging may occur (Level B evidence, Class IIb recommendation).

### Body fluid markers

#### Blood biomarkers

The assessment of bioactive components in bodily fluids such as blood offers the advantages of minimally invasive and high convenience, making it highly suitable for serving as auxiliary clinical indicators for evaluating skeletal muscle aging. In the aging population, early detection of humoral markers can effectively identify individuals with increased high risk for loss of muscle mass and function, thereby preventing the onset and progression of sarcopenia. This consensus aims to recommend biomarkers associated with skeletal muscle aging, thus the strategy for screening fluid biomarkers involves identifying those that are highly correlated with the aging of skeletal muscles (Table S4). These fluid biomarkers offer valuable additional insights for the diagnosis and interventions of skeletal muscle aging. Since these fluid biomarkers may also vary in the context of acute and chronic skeletal muscle diseases, interference from skeletal muscle diseases should be excluded when used in skeletal muscle senescence marker studies.

##### Myokines

Skeletal muscle, the largest endocrine organ in the human body, synthesizes a variety of proteins or peptide substances known as “myokines,” which play crucial roles in maintaining systemic homeostasis through autocrine, paracrine, or endocrine pathways. Myokines have been closely linked to the development of sarcopenia, an age-related condition characterized by a decline in muscle mass and strength. Numerous studies have demonstrated that several inflammation-related myokines, including interleukin-1 (IL-1), interleukin-8 (IL-8), interleukin-18 (IL-18), tumor necrosis factor-α (TNF-α), plasminogen activator inhibitor-1 (PAI-1), matrix metalloproteinase-3 (MMP-3), MMP-9, transforming growth factor-β (TGF-β), and C-reactive protein (CRP), may contribute to the development of sarcopenia [78–80]. Additionally, myokines such as apelin, irisin, myostatin, brain-derived neurotrophic factor (BDNF), and insulin-like growth factor 1 (IGF-1) have also been reported to be closely associated with reductions in skeletal muscle mass and strength [9]. A substantial amount of clinical research has shown that some of these myokines could serve as potential biomarkers for the early diagnosis and treatment of skeletal muscle aging and sarcopenia.

IL-8, also known as CXC chemokine ligand 8 (CXCL8), is one of the most extensively studied chemokines. Initially termed neutrophil-activating factor due to its role in neutrophil degranulation and oxidative burst [81], IL-8 exerts its primary pro-inflammatory physiological functions by binding to its receptor CXCR. A clinical study involving 71 subjects revealed that blood IL-8 levels were significantly higher in sarcopenic individuals compared to healthy controls and were correlated with the severity of sarcopenia [80]. Therefore, IL-8 may be considered a biomarker associated with skeletal muscle aging.

The NLRP3 inflammasome is a key regulator of the innate immune response and induces the maturation and secretion of the downstream effector molecule IL-18, thereby initiating an inflammatory response [82]. A clinical study involving 112 subjects showed that serum IL-18 levels were significantly higher in sarcopenic patients compared to non-sarcopenic patients, while lifestyle interventions (whey protein supplementation + resistance training) significantly improved muscle mass and decreased serum IL-18 levels in sarcopenic patients [83]. Another population-based study, for the first time, simultaneously confirmed the relationship between IL-18 and skeletal muscle aging at both the tissue and serum levels; the study found that not only do aged muscle fibers express high levels of the IL-18 gene, but also that serum IL-18 protein levels were negatively correlated with grip strength in the population [84]. Therefore, IL-18 could be considered a fluid biomarker associated with skeletal muscle aging.

TNF-α is a pleiotropic cellular molecule that plays a central role in inflammation, apoptosis, and immune system development. A meta-analysis based on 80 studies of sarcopenia and frailty showed that community-dwelling sarcopenic and frail individuals had higher blood TNF-α levels compared to age-matched healthy individuals [85]. Therefore, TNF-α could be considered a fluid biomarker associated with skeletal muscle aging.

MMPs, a family of zinc- and calcium-dependent enzymes, degrade extracellular matrix (ECM) proteins. MMPs play a role in regulating skeletal muscle tissue repair and fibrosis, and are crucial for maintaining the structure and function of skeletal muscle myofibrils [86]. A clinical study involving 100 patients with rheumatoid arthritis-associated sarcopenia showed that high blood MMP3 levels were independently associated with sarcopenia [87]. Another study evaluating serum MMP9 levels in 88 sarcopenic patients aged 65 and over showed that serum MMP9/tissue inhibitor of metalloproteinase 1 (TIMP1) had similar performance as a sarcopenia biomarker to the SARC-F questionnaire (AUC = 0.67) [88]. Therefore, MMP3 and MMP9 could be considered as potential biomarkers of skeletal muscle aging.

CRP, a type I acute phase reactant, is a non-specific marker of inflammation and tissue injury. A cross-sectional study of 120 elderly individuals from Iran showed that blood CRP levels were significantly higher in the sarcopenia group compared to the non-sarcopenia group [89]. Another prospective cohort study of 986 elderly individuals showed that higher levels of CRP increased the risk of loss of muscle strength [90]. Therefore, CRP can be considered as a candidate biomarker of skeletal muscle aging, but it needs to be distinguished from infection and injury.

Apelin is an endogenous peptide serves as a ligand for the G protein-coupled receptor APJ. Studies in humans and rodents have shown that circulating apelin levels decrease in an age-dependent manner, while exercise interventions can significantly increase apelin levels [91, 92]. However, a recent small-sample study of elderly individuals over 70 years old showed no significant correlation between blood apelin levels and the incidence of sarcopenia [93]. Therefore, more high-quality basic and clinical studies are needed to confirm the value of blood apelin as a potential biomarker for evaluating skeletal muscle aging.

Irisin is an exercise-induced myokine that exerts its important effects in muscle in an autocrine manner. Numerous studies in humans and mice have shown that circulating irisin levels decrease with age, while exercise can increase circulating irisin levels and improve sarcopenia symptoms [94, 95]. A study of 715 community-dwelling elderly individuals showed that lower levels of circulating irisin were a sensitive marker of muscle weakness and atrophy [96]. However, another study of 143 elderly individuals found no significant correlation between circulating irisin levels and skeletal muscle mass and grip strength parameters associated with sarcopenia [97]. Thus, additional clinical studies are necessary to more accurately assess the effectiveness of irisin as a biomarker for skeletal muscle aging.

Myostatin, a member of the transforming growth factor-β (TGF-β) superfamily, negatively regulates muscle generation in an autocrine mechanism. A cross-sectional study of females across different age groups showed that serum myostatin concentrations increased with age and were negatively correlated with skeletal muscle mass [98]. In addition, a study of 240 individuals of different sexes and ages showed that circulating myostatin levels were positively correlated with age in women, while circulating myostatin levels in men were less affected by age [99]. Given that circulating myostatin levels may be influenced by sex, further validation is needed to determine whether it is suitable for widespread clinical application as a single biomarker of skeletal muscle aging.

BDNF, a member of the neurotrophin family, plays a major role in neuronal growth, differentiation, and plasticity. In skeletal muscle tissue, BDNF regulates the development and differentiation of muscle satellite cells and the survival of motor neurons in a paracrine manner [100, 101]. A study involving 142 elderly participants showed that circulating BDNF levels decreased with age [102]. In contrast, a study of 2522 elderly women aged 65 and over showed a positive correlation between circulating BDNF levels and sarcopenia severity [80]. Thus, the utility of BDNF as a biomarker for skeletal muscle aging requires further investigation.

IGF-1 is an anabolic growth factor that activates the phosphatidylinositol 3-kinase (PI3K)/Akt signaling pathway and playing a crucial role in muscle damage repair and hypertrophy. A case-control study involving 46 healthy subjects and 50 sarcopenic patients showed that blood IGF-1 levels were significantly lower in sarcopenic patients compared to healthy controls [103]. Another cross-sectional analysis of 730 participants aged 65 and over showed that blood IGF-1 levels were significantly lower in sarcopenic patients compared to healthy subjects [104]. However, blood IGF-1 levels are easily influenced by obesity status, and further confirmatory studies are needed to establish its potential as a biomarker for skeletal muscle aging.

##### Other blood protein biomarkers

Hemoglobin (Hb) and serum albumin (ALB) are common clinical biochemical markers that can be used to indicate the occurrence and development of various diseases. Hb, a key component of red blood cells, is responsible for transporting oxygen from the lungs to tissues throughout the body. A study of 730 participants showed that higher Hb levels were significantly associated with faster walking speed and greater grip strength [105]. ALB, the most abundant protein in plasma, also serves as a carrier for nutrients in the blood. A meta-analysis of 14 studies revealed significantly lower serum ALB levels in sarcopenic elderly individuals compared to those without sarcopenia [106]. Additionally, a meta-analysis of 33 160 community-dwelling residents indicated a negative correlation between serum ALB and Hb levels and the severity of sarcopenia and frailty [85]. Therefore, Hb and ALB could serve as markers for assessing skeletal muscle aging.

The ratio of aspartate transaminase (AST) to alanine transaminase (ALT) is a routine blood test indicator used to assess liver damage and infection. Recent clinical studies have found a significant correlation between the AST/ALT ratio and sarcopenia. A Western China Health and Aging Trend (WCHAT) study of 4302 patients showed that the AST/ALT ratio performed well as a biomarker for predicting sarcopenia (AUC = 0.682) [107]. Another cross-sectional study of 2751 community-dwelling elderly people aged 60 and over also found that a higher AST/ALT ratio was associated with an increased risk of sarcopenia [108]. Therefore, the AST/ALT ratio can be considered a potential biomarker for assessing skeletal muscle aging.

Sestrins (SESNs) are highly conserved stress-induced proteins that act as key negative regulators in the oxidative stress and mTORC1 signaling pathways, participating in the sensing and integration of nutritional and environmental cues, and influencing cell growth, metabolism, and inflammation. Sestrin 1–3 are considered to be major factors in regulating skeletal muscle mass and metabolism [109, 110]. Recent studies have shown that Sestrin 1–3 play a crucial role in preventing muscle atrophy due to aging and that they can be secreted into the blood [111–115]. Given these findings, Sestrin 1–3 have the potential to be used as biomarkers for assessing skeletal muscle aging. However, more clinical studies are needed to establish their effectiveness and specificity in this regard.

##### Hormone-related biomarkers

Testosterone exerts protective effects on skeletal muscle, including anti-catabolic and anti-inflammatory properties. Studies have shown that testosterone levels gradually decline with age in men, a change that is closely associated with decreases in muscle mass and strength [116]. In a cross-sectional study involving 730 participants, a decrease in blood bioavailable testosterone levels was found to be independently associated with the occurrence of sarcopenia [104]. However, due to its susceptibility to obesity and chronic diseases, testosterone may not be suitable as an independent biomarker of skeletal muscle aging in men.

Dehydroepiandrosterone (DHEA), a steroid secreted by the adrenal glands, serves as a precursor to both estrogen and androgen. DHEA levels peak between the ages of 20 and 30 and then gradually decline with age [117]. A cross-sectional study of 478 women aged 50–90 years showed that serum DHEA was correlated with muscle strength and function [118]. However, another study based on 100 community-dwelling elderly individuals showed that DHEA was not significantly correlated with muscle mass or strength in either men or women [119]. Therefore, further clinical studies are needed to clarify the effectiveness of serum DHEA as a biomarker for assessing skeletal muscle aging.

The biologically active form of vitamin D, 25(OH)D, influences skeletal muscle function by binding to the vitamin D receptor (VDR). Mendelian randomization studies have shown an association between serum 25(OH)D levels and the risk of sarcopenia [120]. A study of 83 patients with hip fracture-related sarcopenia found that bioavailability of 25(OH)D levels was significantly lower in the sarcopenia group compared to the non-sarcopenia group [121]. However, another cross-sectional study of 131 elderly individuals aged 60–85 years did not find a significant correlation between serum 25(OH)D levels and sarcopenia [122]. Thus, additional clinical studies are required to further evaluate the effectiveness of 25(OH)D in the blood as a biomarker for skeletal muscle aging.

Skeletal muscle is the primary tissue for glucose uptake under the stimulation of insulin and plays a crucial role in glucose metabolic balance. Studies have shown a close link between reduced skeletal muscle mass and insulin resistance (IR) [123]. In the Western China Health and Aging Trend study involving 4302 participants, fasting insulin levels showed good performance as a biomarker for predicting the occurrence of sarcopenia (AUC = 0.686) [107]. However, blood insulin levels are easily affected by obesity status, and more clinical studies are still needed to confirm its practical utility.

Adiponectin is an insulin-sensitizing hormone that promotes fatty acid oxidation and glucose uptake in skeletal muscle cells, thereby enhancing the ability of insulin to suppress gluconeogenesis. A meta-analysis of seven cross-sectional studies showed a significant association between sarcopenia and higher adiponectin levels [124]. Additionally, a retrospective study of 132 cardiovascular disease patients with sarcopenia found that adiponectin was an independent predictor of sarcopenia [125]. However, blood adiponectin levels can be influenced by obesity, and more clinical studies are still needed to evaluate its practical utility.

##### Serum creatinine/cystatin C (Cr/Cysc) levels

Cystatin C (CysC) is an ideal biomarker for assessing glomerular filtration rate in clinical settings, as its levels are not affected by factors such as gender and diet. Creatinine (Cr) is the end product of the metabolism of creatine and phosphocreatine in muscle, and its levels are primarily affected by muscle mass, meat intake, and kidney function. In the case of stable kidney function, the main factor determining fasting serum Cr/CysC is considered to be skeletal muscle mass [126]. Numerous clinical studies have evaluated the diagnostic value of serum Cr/CysC for sarcopenia, finding sensitivities ranging from 41% to 95% and specificities ranging from 38% to 94% [127]. Therefore, serum Cr/CysC has good diagnostic accuracy in assessing sarcopenia and can be recommended as a biomarker of skeletal muscle aging, while kidney function indicators should also be included in the assessment.

##### Amino acids and other metabolites

Protein carbonyls, a major marker of oxidative protein damage, represent proteins that have been oxidized and modified by numerous reactive oxygen species (ROS, lipid peroxidation-derived aldehydes, and reducing sugars). A cross-sectional study of 672 women aged 65 and over showed that high serum levels of protein carbonyls were independently associated with low grip strength in community-dwelling elderly women [128]. Another study of 88 hemodialysis patients with sarcopenia found that serum protein carbonyl levels were significantly correlated with the severity of sarcopenia [129]. Based on these findings, blood protein carbonyl levels have the potential to be used as a biomarker for assessing skeletal muscle aging, but more clinical studies are needed to evaluate their practical utility.

##### Other potential blood biomarkers

Advanced glycation end products (AGEs) are a heterogeneous substances of non-enzymatic products derived from the reaction of glucose or other sugar derivatives with proteins or lipids and serve as biomarkers of aging and disease. Elderly individuals and those with diabetes often suffer from musculoskeletal diseases, which is associated with the accumulation of AGEs. A systematic review and meta-analysis summarizing 14 cross-sectional studies and one prospective study showed that circulating AGEs were negatively correlated with muscle strength and physical function [130]. AGEs have the potential to be a candidate biomarker for skeletal muscle aging, but more clinical studies are needed to evaluate their practical utility.

microRNA-486 (miRNA-486) is highly expressed in skeletal muscle and promotes myocyte differentiation by directly targeting Pax7 [131]. microRNA-146a (miRNA-146a) is an anti-inflammatory microRNA (miRNA) that negatively regulates the inflammatory response by inactivating cytoplasmic NF-κB through the activation of TNF receptor-associated factor 6 (TRAF6) and IL-1R-associated kinase (IRAK-1). A cross-sectional study of 597 community-dwelling elderly individuals showed that plasma levels of miRNA-486 and miRNA-146a were significantly lower in sarcopenic patients, and they performed well as predictors of sarcopenia (AUC values of 0.708 and 0.676, respectively) [132]. miRNA-486 and miRNA-146a have the potential to be used as biomarkers for assessing skeletal muscle aging, but more clinical studies are needed to validate their performance.

Circulating cell-free mitochondrial DNA (ccf-mtDNA) is approximately 50–200 bp fragments of degraded DNA found in plasma, composed of free mitochondria and a small amount of nuclear DNA. Mitochondrial DNA is thought to promote inflammation during aging. In a clinical study of 97 elderly individuals, circulating free cell mtDNA levels were significantly higher in sarcopenic patients compared to healthy controls, and circulating mtDNA showed good diagnostic efficacy as a biomarker for sarcopenia (AUC value of 0.726) [133]. Circulating mitochondrial mtDNA is a potential biomarker for assessing skeletal muscle aging, but more clinical studies are needed to corroborate this.

#### Urinary biomarkers

In addition to serum Cr/CysC levels, urinary Cr level has been considered a more reliable indicator for assessing muscle mass. The urinary Cr measurement based on the oral isotope D3-creatine dilution method is a technique for quantifying skeletal muscle, with results reflecting the size of the body’s creatine pool. This method offers a more direct assessment of skeletal muscle mass compared to conventional methods such as bioelectrical impedance analysis (BIA) and dual-energy X-ray absorptiometry (DXA) [3, 134]. A cohort study of 1382 men (mean age 84.2 years) showed that the D3-creatine dilution method was more closely associated with outcomes such as physical performance and activity in men than DXA lean mass [135]. Another retrospective analysis showed that reduced Cr excretion in ICU patients was associated with estimated skeletal muscle mass [136]. Thus, the D3-creatine dilution method for urinary Cr measurement can be considered a biomarker for assessing skeletal muscle aging. However, since creatine and phosphocreatine are filtered by the glomeruli, renal function indicators should also be included in the assessment.

#### Gut microbiota biomarkers

##### Microbial community structure and species variation

Aging brings changes to our gut microbiota, where most communities are typically made up of just “five phyla”, including *Bacteroidetes*, *Firmicutes*, *Actinobacteria*, *Proteobacteria*, and *Verrucomicrobia*. Notably, it shifts the balance of certain bacteria, like *Sutterella* and *Barnesiella* species, which are linked to a decline in muscle function [137]. Studies have found that the levels of *Bifidobacterium* decrease with age. Therefore, the age-related reduction in intestinal *Bifidobacterium* content may underlie the increase in circulating endotoxins, which have been shown to induce skeletal muscle atrophy [138]. Not only that, but in the intestines of elderly individuals, there is typically a decrease in bacteria that produce short-chain fatty acids, such as *Roseburia* and *Faecalibacterium*, while there is an increase in oxygen-tolerant and pathogenic bacteria [139]. These changes can lead to an imbalance in the microbial community, which in turn can trigger the onset of various age-related diseases, including sarcopenia. Most studies have found that both the diversity and richness of the gut microbiota are reduced in individuals with sarcopenia.

Observational studies reveal that the gut microbiome differs among individuals. In frail elderly individuals, there is a notable decline in *Lactobacillus* species (such as *Lactobacillus acidophilus*), *Bifidobacterium*, and other *Lactobacillus* types, accompanied by an increase in *Enterobacteriaceae* [140]. The gut microbiota diversity in individuals with sarcopenia is significantly lower compared to that in the control group. In another study involving 27 sarcopenia cases and 60 controls, it was found that the abundance of butyrate-producing bacteria, such as *Lachnospira*, *Fusicantenibacter*, *Roseburia*, *Eubacterium*, and *Lachnoclostridium* was significantly lower in the sarcopenia group, while the abundance of *Lactobacillus* was higher [141]. Another study found that the abundance of bacteria associated with short-chain fatty acid production, such as *Faecalibacterium prausnitzii* and *Roseburia inulinivorans*, was lower in sarcopenia cases compared to non-sarcopenia controls [142]. Our research team, through metagenomic analysis of the gut microbiota in 141 sarcopenia patients and 142 non-sarcopenia individuals, discovered that at the genus/species level, the relative abundance of *Bacteroides vulgatus*, *Bifidobacterium*, *Roseburia*, and *Faecalibacterium prausnitzii* were higher in sarcopenia patients, while the abundance of *Eubacterium rectale* and *Prevotella copri* was lower in sarcopenia patients [143]. Another study, which analyzed the gut microbiota using 16S rRNA sequencing in 141 sarcopenia patients and 1276 non-sarcopenia individuals, found that the abundance of *Desulfovibrio piger*, *Clostridium symbiosum*, *Hungatella effluvii*, *Bacteroides fluxus*, *Absiella innocuum*, and *Clostridium citroniae* was significantly increased in sarcopenia patients compared to non-sarcopenia controls. Furthermore, these increases were positively correlated with the severity of sarcopenia [144].

Previous studies have also analyzed the gut microbiota in secondary sarcopenia. Zhou et al. conducted a study comparing the gut microbiota of 30 patients with maintenance hemodialysis (MHD) and 30 patients with sarcopenia and MHD. They found that the abundance of *Tyzzerella_4* was increased in patients with sarcopenia and MHD, while the abundance of *Megamonas*, *Coprococcus_2*, and *uncultured_bacterium_f_Muribaculaceae* was decreased [145]. Margiotta et al. conducted a study on the gut microbiota of 18 patients with sarcopenia and chronic kidney disease (CKD) and 45 patients with non-sarcopenia and CKD. They found that the abundance of *Micrococcaceae*, *Verrucomicrobiaceae*, *Megasphaera*, *Rothia*, *Veillonella*, *Akkermansia*, and *Coprobacillus* was increased in patients with CKD and sarcopenia [146]. Ponziani et al. analyzed the gut microbiota of 50 patients with sarcopenia and liver cirrhosis and 50 patients with non-sarcopenia and liver cirrhosis. They found that the abundance of *Eggerthella* and *Klebsiella* was increased, while the abundance of *Methanobrevibacter*, *Prevotella*, and *Akkermansia* was decreased in patients with liver cirrhosis and sarcopenia [147]. Additionally, Ren et al. conducted a study on the gut microbiota of 30 controls, 30 patients with liver cirrhosis and sarcopenia, and 30 patients with liver cirrhosis and non-sarcopenia. They discovered that the relative abundance of *Escherichia coli*, *Peptostreptococcus stomatis*, and *Bacteroides uniformis* showed the most significant association with L3 SMI [148]. Furthermore, Picca et al. studied the gut microbiota of 17 controls and 18 patients with muscle loss and frailty, finding that the abundance of *Oscillospira* and *Ruminococcus* was increased, while the abundance of *Barnesiellaceae* and *Christensenellaceae* was decreased in patients with sarcopenia and frailty [149].

Animal studies have found that a decrease in the abundance of butyrate-producing bacteria, such as *Roseburia* and *Clostridium XIVb*, in the gut of ghrelin-knockout (Ghrl^−/−^) mice during their early years may exacerbate muscle loss in aging [150]. Research has shown that *Lactobacillus* and *Bifidobacterium* strains can help restore age-related muscle loss. Transplantation of *Bacteroides fragilis* into the gut of germ-free mice resulted in higher muscle mass and muscle function compared to germ-free mice [137]. Nine probiotic supplements, including *Saccharomyces boulardii* (SB), *Lactobacillus bulgaricus* LC122 (LC122), *Bifidobacterium longum* BL986 (BL986), *Lactobacillus paracasei* PS23 (LPPS23), *Lactobacillus salivarius* SA-03 (SA-03), *Lactobacillus plantarum* TWK10 (LP10), *Bifidobacterium longum* OLP-01 (OLP-01), *Lactobacillus reuteri*, and *Lactobacillus gasseri*, have been reported to be beneficial for muscle growth and function [151–157].

#### Changes in metabolites

The gut microbiota produces substances such as branched-chain amino acids (BCAAs), tryptophan, short-chain fatty acids (SCFAs), bile acids, urolithin A, lipopolysaccharide (LPS), peptidoglycan, and indoxyl sulfate through the metabolism of nutrients. These substances participate in multiple signaling pathways and exerting a beneficial or bad effect on the anabolism of skeletal muscle [155–179].

##### BCAAs

The gut microbiota can synthesize some BCAAs, which include leucine, isoleucine, and valine, accounting for 14%–18% of all amino acids in skeletal muscle proteins. BCAAs play a crucial physiological role in the regulation of protein synthesis, metabolism, food intake, and aging [158]. BCAAs, especially leucine, can promote muscle protein synthesis and inhibit muscle protein degradation. Furthermore, studies have shown that leucine can promote protein synthesis by reducing the phosphorylation of eIF2a and increasing the binding of eIF4E and eIF4G through the phosphorylation of mammalian target of rapamycin (mTOR, a protein kinase), ribosomal protein S6 kinase (S6K1), and eukaryotic initiation factors, as well as the phosphorylation of 4E-binding protein-1 [159]. Another study demonstrated that BCAAs can promote the phosphorylation and activation of p70S6 kinase and 4EBP1 in skeletal muscle, which can promote muscle protein synthesis [160]. p70S6 kinase and 4EBP1 are downstream components of the mTOR signaling pathway, controlling RNA translation and protein synthesis, and are considered key points leading to muscle hypertrophy. A recent meta-analysis of 35 original studies suggested that supplementation with BCAA-rich nutrients may have a beneficial impact on muscle mass and strength in the elderly [161]. However, the maintenance of muscle protein synthesis rates is related to the additional supplementation of sufficient essential amino acids, so a decrease in the intestinal synthesis and metabolism rate of BCAAs may be associated with the occurrence of sarcopenia.

Tryptophan (Trp) is an essential aromatic amino acid. Although it is present in the smallest amounts in proteins and cells, it serves as a biosynthetic precursor for a large number of microbial and host metabolites [162]. Trp is metabolized in the gut mainly through three pathways: (i) the production of indole and its derivatives by the gut microbiota, such as indole-3-aldehyde (IAld), indole-3-acetic acid (IAA), indole-3-propionic acid (IPA), indole-3-acetaldehyde (IAAld), and indoleacrylic acid; (ii) the production of kynurenine (Kyn) and its downstream products through the Kyn pathway; and (iii) the production of 5-hydroxytryptamine (5-HT) through the serotonin pathway [163]. As an essential amino acid, Trp plays an important role in maintaining muscle mass during individual development and aging. In the elderly, serum Trp levels decrease with age, and animals deficient in Trp exhibit significant muscle atrophy [164]. Supplementation with Trp in aged mice increased the expression of muscle-derived IGF-1 and gene expression in the eIF4/p70s6k/mTor pathway, and alleviated muscle loss caused by dietary protein deficiency. *In vitro*, Trp stimulated the expression of myogenic markers MyoD, myogenin, and myosin heavy chain [165].

##### SCFAs

The colonic microbiota can produce SCFAs, which mainly include acetic acid, propionic acid, isobutyric acid, butyric acid, isovaleric acid, valeric acid, isocaproic acid, and caproic acid. Among them, acetic acid, propionic acid, and butyric acid account for more than 95% of the total SCFAs. The anaerobic bacteria involved in glycolysis mainly include some strains of *Bifidobacterium*, *Lactobacillus*, *Bacteroides*, and *Fusobacterium* [166]. Studies have found that SCFAs in gut microbiota metabolites can target muscle mitochondria, promote mitochondrial biosynthesis, and regulate fatty acid metabolism by binding to fatty acid receptors 2 and 3 in skeletal muscle cells [167]. A study found that treatment with butyrate in C57/BL6 female mice starting from the 16th month could completely or partially improve hindlimb muscle atrophy by increasing muscle fiber cross-sectional area and preventing muscle fat accumulation in mice [168]. Therefore, butyrate can increase mitochondrial biosynthesis in skeletal muscle and whole-body oxygen consumption, promote ATP production and muscle protein synthesis, increase muscle glycogen levels, reduce oxidative stress and cellular apoptosis, and alter antioxidant enzyme activity to promote an increase in muscle mass and muscle function.

Bile acids: Intestinal microbial enzymes produce unconjugated bile acids and secondary bile acids through dissociation and dehydroxylation. Studies have shown that levels of 12α-OH bile acids (such as deoxycholic acid) are associated with a decrease in skeletal muscle volume, while levels of non-12α-OH bile acids (such as chenodeoxycholic acid) are positively correlated with skeletal muscle volume [169]. Additionally, bile acids can bind to the G-protein-coupled receptor TGR5, leading to increased production of cyclic adenosine monophosphate (cAMP), which in turn increases the activity and oxygen consumption of type 2 iodothyronine deiodinase (D2) in brown adipocytes and human skeletal muscle cells. Activation of D2 can increase T3 levels in skeletal muscle cells, thereby promoting skeletal muscle development and regeneration [170]. Animal experiments have also found that low doses of ursodeoxycholic acid can reduce muscle tissue loss in the gastrocnemius, extensor digitorum longus, and soleus muscles, while high doses of ursodeoxycholic acid can reduce weight loss and decrease mortality in tumor-bearing mice [171]. Tauroursodeoxycholic acid can also increase insulin sensitivity in muscle tissue and reduce fat infiltration [172]. Studies have shown that bile acids increase skeletal muscle protein synthesis and inhibit muscle atrophy through the FXR-FGF15/19 pathway [173, 174]. Multiple data indicate that increasing circulating levels of FGF19 through pharmacological treatment or transgenic over-expression can increase muscle protein synthesis, increase muscle fiber size, improve skeletal muscle mass, prevent muscle atrophy, and simultaneously increase energy expenditure, reduce fat mass, and improve lipid and glucose homeostasis in various mouse models [175–178].

##### Urolithin A

Urolithin A, a metabolite produced by the gut, can repair mitochondrial and muscle function during aging. Studies have found that supplementing the diet of mice with urolithin A can delay the onset of Duchenne muscular dystrophy (DMD). A study was conducted among middle-aged adults (*n* = 88) aged 40 to 64 years in London, Ontario, Canada. Participants were randomly assigned to receive daily supplements of 500 mg, 1000 mg of urolithin A, or a placebo for four consecutive months. Muscle strength, physical performance tests, skeletal muscle biopsies, and biomarkers of healthy mitochondrial function and inflammation in plasma were assessed at baseline, 2 months, and 4 months. It was found that after 4 months of daily urolithin A intake, muscle strength significantly improved by 12%. These results further demonstrate that urolithin A is beneficial for muscle and mitochondrial health, and is safe, and well-tolerated [179].

##### LPS

LPS is a unique component of the cell wall of Gram-negative bacteria, which helps bacteria maintain their structure and resist antibiotics, the complement system, and other environmental stresses. LPS is released into the intestinal environment and enters the bloodstream when bacteria die and lyse. When high concentrations of LPS enter the bloodstream, they can cause a series of severe pathophysiological reactions such as fever, coagulation, and shock by disrupting the host’s immune system, complement system, and coagulation system, which is referred to as endotoxemia. Furthermore, increased LPS levels can damage the tight junctions of intestinal epithelial cells and increase intestinal permeability, triggering a series of inflammatory factor responses and promoting the development of sarcopenia [180].

##### Peptidoglycan

Peptidoglycan, found in the cell walls of most bacteria, is a mechanically strong, multi-layered, net-like macromolecular structure formed by the polymerization of N-acetylglucosamine, N-acetylmuramic acid, and short amino acid peptides. It constitutes the basic framework of bacterial cell walls. The peptidoglycan content can reach 50%–80% in the cell walls of Gram-positive bacteria, but only accounts for 5%–20% in the cell walls of Gram-negative bacteria. The thickness and structure of peptidoglycan are the main factors distinguishing Gram-positive bacteria from Gram-negative bacteria. The conserved structure of peptidoglycan, with its bacterial-specific molecular characteristics, has evolved over time to become a bacterial-specific signal recognized by the host’s immune system, serving as an immunostimulant for the animal immune system. Peptidoglycan can stimulate mononuclear phagocytes and endothelial cells to release small inflammatory molecules such as TNF-α, interleukins, and interferons, promoting the development of sarcopenia [181].

##### Indoxyl sulfate

Indoxyl sulfate, produced by the intestinal microbiota, accelerates the cascade reaction of skeletal muscle atrophy by activating the ROS–ERK axis and the JNK–MAFbx axis. It upregulates the expression genes of myostatin and atrogin-1, reduces mitochondrial energy, and increases the production of inflammatory cytokines, thereby promoting the development of sarcopenia [182]. Another study found that after treating C2C12 muscle cells with indoxyl sulfate, both the morphology and diameter of the muscle cells were significantly reduced [183].

#### Recommendations

(1) Plasma IL-18, CRP, Hb, ALB, and AST/ALT: Existing clinical evidence suggests that they are associated with skeletal muscle aging but have not been clinically applied. It is recommended to use them as humoral biomarkers associated with skeletal muscle aging, while excluding the influence of other underlying diseases (Level B evidence, Class IIb recommendation).
(2) Plasma Cr/CysC: Extensive clinical evidence suggests that they are associated with skeletal muscle aging but have not been clinically applied. They can be considered as humoral biomarkers associated with skeletal muscle aging, while renal function indicators should be included in the assessment (Level B evidence, Class IIa recommendation).
(3) Plasma IL-8, TNFα, MMP3, MMP9, miRNA-486, miRNA-146a, AGE, mtDNA, and carbonylated proteins: Limited clinical data suggests that they are associated with skeletal muscle aging but have not been clinically applied. They can be considered as candidate humoral biomarkers for sarcopenia, warranting larger-scale clinical validation. Given their non-specificity to skeletal muscle, a comprehensive assessment considering overall bodily aging is necessary (Level C evidence, Class IIb recommendation).
(4) Plasma Sestrin 1–3 and other highly skeletal muscle-specific markers: Preliminary data suggest their relevance to sarcopenia of aging, but further clinical research is needed for confirmation. They can be considered as candidate humoral biomarkers for sarcopenia, deserving of larger-scale clinical validation (Level C evidence, Class IIb recommendation).
(5) The detection of urinary Cr levels based on the D3-creatine dilution method can accurately assess skeletal muscle mass relative to conventional detection methods, such as BIA and DXA. It is recommended as a humoral biomarker associated with skeletal muscle aging, while renal function indicators should be included in the assessment (Level B evidence, Class IIb recommendation).
(6) The abundances of *Bifidobacterium*, *Lactobacillus*, *Roseburia*, *Prevotella*, and *Bacteroides* in the intestinal microbiota decrease sharply, which may suggest potential skeletal muscle aging (Level C evidence, Grade III recommendation). It is not recommended to use changes in the structure and species composition of the intestinal microbiota alone as markers of skeletal muscle aging.
(7) The levels of BCAAs, tryptophan, SCFAs, and secondary bile acids in intestinal metabolites decrease sharply, which may suggest potential skeletal muscle aging (Level C evidence, Grade III recommendation). It is not recommended to use changes in the levels of intestinal metabolites alone as markers of skeletal muscle aging.

## Assessment of skeletal muscle aging, construction of prediction models

Skeletal muscle aging involves changes at multiple levels, including molecules, cells, organs, and population dimensions. To accurately assess the degree of skeletal muscle aging, it is necessary to integrate information from multi-dimensional and multi-scale perspectives. At present, predicting the biological age of skeletal muscle remains an unresolved challenge. This consensus statement elaborates on skeletal muscle aging biomarkers from functional, structural, and humoral perspectives of skeletal muscle, aiming to provide quantitative indicators for the precise assessment of skeletal muscle biological age. Actually, clinical assessments of skeletal muscle function in the elderly population are primarily conducted through functional tests and imaging techniques. Current challenges: low accuracy of prediction and evaluation, as well as high difficulty in practical application. In the future, a comprehensive consideration should be given from multiple dimensions, such as “function”, “imaging”, and “humoral” dimensions, to develop a precise and practical combined protocol. It is noteworthy that skeletal muscle aging begins earlier than other organs do, such as a gradual decrease in human skeletal muscle mass and function starting around the age of 30–40. This implies the limitations of existing methods for assessing skeletal muscle aging.

Population cohort studies are powerful models for identifying disease risk factors. To date, our country has also established several cohorts for sarcopenia research, such as the WCHAT and the Xiangya Sarcopenia study. These studies have advanced research on the etiology and pathogenesis of sarcopenia in China. Nevertheless, there is currently no mature model that can accurately predict the occurrence of sarcopenia. Future research needs to integrate physiological information and specific biomarkers from existing sarcopenia cohort populations to construct more precise prediction models, which are of great significance for the diagnosis of sarcopenia and the prediction of skeletal muscle aging.

With the development of high-throughput technologies such as genomics, transcriptomics, DNA methylomics, metabolomics, and proteomics have been widely applied to aging research. The development and maturation of emerging single-cell/single-nucleus sequencing, spatial transcriptomics, and spatial metabolomics technologies provide the possibility of discovering more specific biomarkers that indicate skeletal muscle aging. Therefore, future research should be dedicated to establishing a multi-dimensional “aging clock” that spans macro and micro levels, incorporating various parameters to accurately evaluate and predict the degree of human skeletal muscle aging.

## Conclusion and future perspectives

### Recommendation of skeletal muscle aging biomarkers

Based on the discussion experts, skeletal muscle aging biomarkers are recommended from three dimensions: function, structure, and body fluids, which will be validated in cohort studies in the future. In addition, based on the variations in different biomarkers, personalized and effective treatment plans can be provided for patients in the future.

Functional biomarkers: grip strength, physical performance (walking speed, 6MWT, 400MWT, TUG, and SPPB) and HOMA-IR. Structural biomarkers: skeletal muscle mass, number and area of skeletal muscle fibers, adipose deposition, immune cell infiltration, fibrosis, and degeneration of the neuromuscular junction. Body fluid biomarkers: Skeletal muscle-related markers (plasma (Cr/Cysc), CRP, Hemoglobin, Albumin, AST/ALT, TNFα, MMP3, MMP9, Sestrin 1–3, miRNA-486, miRNA-146a, AGE, mitochondrial DNA, and carbonylated proteins, urinary Cr) (Table 3).

**Table 3.** Key recommended biomarkers of skeletal muscle aging

Dimension	Biomarker	Test method	COR	LOE
Functional markers	Grip strength	Dynamograph	I	A
	Physical performance	Walking speed, 6MWT, 400MWT, TUG, SPPB	I	A
	Insulin resistance index	Spectrophotometry and ELISA	IIa	A
Imaging markers	Skeletal muscle mass	DXA, CT, BIA, MRI, US	I	A
	Number and area of skeletal muscle fibers	DXA, CT, MRI, US	I	A
	Adipose deposition	MRI, CT	IIb	B
	Degeneration of the neuromuscular junction	EMG, sEMG	IIb	B
	Fibrosis	MRI, CT	IIb	C
Body fluid markers	Cr/CysC	ELISA	IIa	B
	IL-18, CRP, Hb, ALB, AST/ALT, Urinary creatinine	ELISA	IIb	B
	IL-8, TNFα, MMP3, MMP9, Sestrin 1–3, miRNA-486, miRNA-146a, AGE, mtDNA, carbonylated proteins	ELISA	IIb	C

### The working route map of our skeletal aging biomarker research

Through expert consensus, these skeletal muscle biomarkers will be further validated in population cohorts in the future. Establishing a link between basic research and clinical research will accelerate the research process for precise diagnosis and intervention of skeletal muscle aging or sarcopenia, contributing to the healthy aging of human skeletal muscles.

The framework of China’s skeletal muscle aging biomarker research includes (i) Promoting the construction of “large-region, large-sample skeletal muscle aging” cohorts in China, establishing a large-scale, multi-dimensional skeletal muscle sample library, and cross-validating with various detection methods; (ii) Painting a portrait of skeletal muscle aging from multiple dimensions, integrating information on population physiology, biomarkers, lifestyle, etc., to establish a set of skeletal muscle biological age measurement systems and predictive models that multi-dimensional parameters. (iii) Clinical research to verify the effectiveness and practicality of biomarkers in diagnosis and treatment, and to formulate guidelines for skeletal muscle aging assessment and intervention based on biomarkers; (iv) Establishing research consortium and networks, combine industry, academia, and research to promote the establishment and application of research guidelines and standards, organizing interdisciplinary seminars, sharing the latest research results and ideas, engaging in international exchange and cooperation, thereby promoting skeletal muscle aging research in China and improving the level of skeletal muscle health.

## Supplementary Material

lnaf001_suppl_Supplementary_Data

lnaf001_suppl_Supplementary_Tables_S1-S4
