# Supplementary material for: A framework of biomarkers for skeletal muscle aging: a consensus statement by the Aging Biomarker Consortium
Source: Life Med. 2025 Jan 26;3(6):lnaf001. doi: 10.1093/lifemedi/lnaf001 (PMC11851484; doi:10.1093/lifemedi/lnaf001)
Supplement: lnaf001_suppl_Supplementary_Tables_S1-S4 [file lnaf001_suppl_supplementary_tables_s1-s4.pdf]

Supplementary table 1. 6 Minute walk test (6MWT)

Project	Content	Evaluation criteria	Score
6 minute walk test	Six-minute walk distance	< 150 m	Severe abnormality
		151–300 m	Moderate abnormality
		301–450 m	Mild abnormality
		> 450 m	Normal

Supplementary table 2. Timed up and go test

Project	Content	Evaluation criteria	Score
Timed up and go test	Timed up and walk	≥ 30 s	Severe abnormality
		20–29 s	Moderate abnormality
		10–19 s	Mild abnormality
		< 10 s	Normal

Supplementary table 3. Scoring for short physical performance battery (SPPB)

Test	Content	Evaluation criteria	Score
Gait speed test	4-meter gait speed	< 4.82 s	4 points
		4.82–6.20 s	3 points
		6.21–8.70 s	2 points
		> 8.70 s	1 point
		Unable to complete	0 points
Balance test	Side-by-side stand	≥ 10 s	1 point
		<10 s or not attempted	0 points
	Semi-tandem stand	≥ 10 s	1 point
		< 10 s or not attempted	0 points
	Tandem stand	≥ 10 s	2 points
		3–9.99 s	1 point
		< 3 s or not attempted	0 points
Repeated chair stands	Time to complete five stands	< 11.19 s	4 points
		11.20–13.69 s	3 points
		13.70–16.69 s	2 points
		> 16.7 s	1 point
		Unable to complete or > 60 s	0 points

Supplementary table 4. Recommended biomarkers of skeletal aging

Dimension	Biomarker	Test method	COR	LOE
Humoral	IL-8	Plasma/ELISA	IIb	C
	IL-18	Plasma/ELISA	IIb	B
	TNFα	Plasma/ELISA	IIb	C
	MMP3	Plasma/ELISA	IIb	C
	MMP9	Plasma/ELISA	IIb	C
	CRP	Plasma/ELISA	IIb	B
	Hb	Plasma/ELISA	IIb	B
	ALB	Plasma/ELISA	IIb	B
	Sestrin 1-3	Plasma/ELISA	IIb	C
	AST/ALT	Plasma/ELISA	IIb	B
	Cr/CysC	Plasma/ELISA	IIa	B
	miRNA-486	Plasma/ELISA	IIb	C
	miRNA-146a	Plasma/ELISA	IIb	C
	AGE	Plasma/ELISA	IIb	C
	mtDNA	Plasma/ELISA	IIb	C
	Protein carbonyls	Plasma/ELISA	IIb	C
	Urinary creatinine	Urinary/ D3-creatine dilution method	IIb	B
